# Computational Efficient Motion Planning Method for Automated Vehicles Considering Dynamic Obstacle Avoidance and Traffic Interaction

**DOI:** 10.3390/s22197397

**Published:** 2022-09-28

**Authors:** Yuxiang Zhang, Jiachen Wang, Jidong Lv, Bingzhao Gao, Hongqing Chu, Xiaoxiang Na

**Affiliations:** 1 The State Key Laboratory of Automotive Simulation and Control, Jilin University, Changchun 130025, China; 2 The College of Computer Science and Technology, Jilin University, Changchun 130015, China; 3 The Utopilot SAIC MOTOR, Shanghai 200438, China; 4 The Clean Energy Automotive Engineering Center, Tongji University, Shanghai 201804, China; 5 School of Automotive Studies, Tongji University, Shanghai 201804, China; 6 The Department of Engineering, University of Cambridge, Cambridge CB2 1PZ, UK

**Keywords:** autonomous vehicles, trajectory planning, model predictive control

## Abstract

In complex driving scenarios, automated vehicles should behave reasonably and respond adaptively with high computational efficiency. In this paper, a computational efficient motion planning method is proposed, which considers traffic interaction and accelerates calculation. Firstly, the behavior is decided by connecting the points on the unequally divided road segments and lane centerlines, which simplifies the decision-making process in both space and time span. Secondly, as the dynamic vehicle model with changeable longitudinal velocity is considered in the trajectory generation module, the C/GMRES algorithm is used to accelerate the calculation of trajectory generation and realize on-line solving in nonlinear model predictive control. Meanwhile, the motion of other traffic participants is more accurately predicted based on the driver’s intention and kinematics vehicle model, which enables the host vehicle to obtain a more reasonable behavior and trajectory. The simulation results verify the effectiveness of the proposed method.

## 1. Introduction

Due to the complex driving scenarios and difficulty in accurately predicting the behavior of the surrounding vehicles, the automated vehicles need to adapt to great complexity and dynamics in real traffic [1,2]. Therefore, advanced motion planning algorithms should help the agent behave reasonably and respond adaptively in dynamic and complex driving scenarios with computational efficient and reliable control [3].

### 1.1. State-of-the-Art Review and Challenges

The performance of motion planning methods is closely related to the trajectory prediction of other traffic participants, behavior planning, and trajectory generation. As automated vehicles will frequently interact with other traffic participants, trajectory prediction will influence behavior planning and trajectory generation modules. The motion of other environment vehicles is predicted with constant longitudinal velocity or acceleration [4]. Such a prediction is inaccurate and will decrease the reasonability of behavior planning and trajectory generation modules [5,6]. Thus, more accurately predicted trajectories will promote the performance of motion planning.

In behavior planning aspects, the machine learning-based or model-based methods usually decide a human-like behavior, like lane-change and obstacle avoidance, which contains a wide range of trajectories. To ensure absolute safety, decision-making methods are always conservative. Thus, more complex behaviors should be generated in decision-making. POMDP can generate a more abundant behavior [7,8]. The road is divided into several segments and the points are connected to represent different behavioral decisions [9,10].

Regarding trajectory generation, current methods can be divided into graph-search-based methods [11], incremental search [12], interpolating curve methods [13] and numerical optimization [14,15]. Model Predictive Control (MPC) is widely used because it can explicitly deal with constraints to ensure safety with consideration of traffic interaction not only at the current time step but also in the predictive horizon [16,17]. As for the control model in MPC, the dynamic vehicle model is added to the kinematic model to realize stable motion control and enrich driving behaviors [18,19].

The high-performance motion planning methods should be computationally efficient and enable automated vehicles to behave reasonably and respond adaptively in dynamic and complex scenarios. First, the host vehicle will interact with the surrounding vehicles while driving on the road inevitably. The motion prediction of environment vehicles needs to be predicted in the planning horizon, which enables the host vehicle to behave reasonably and adapt to the dynamic traffic environment. Meanwhile, the driving process contains a large span in both time and space, which means precise driving behavior will cause huge calculations. To balance the calculation time and planning performance, the problem in the model formulation aspect should be simplified without losing reasonability.

To further promote the reasonability of motion planning, except for the dynamic vehicle model, variational velocity can be considered in the trajectory generation. Thus, the control model will change from the linear model to the nonlinear model [20]. Such a control model increases the time for online solving, which needs an additional fast solving algorithm to realize online calculation [21,22,23].

### 1.2. Work and Contributions

**Figure 1:**
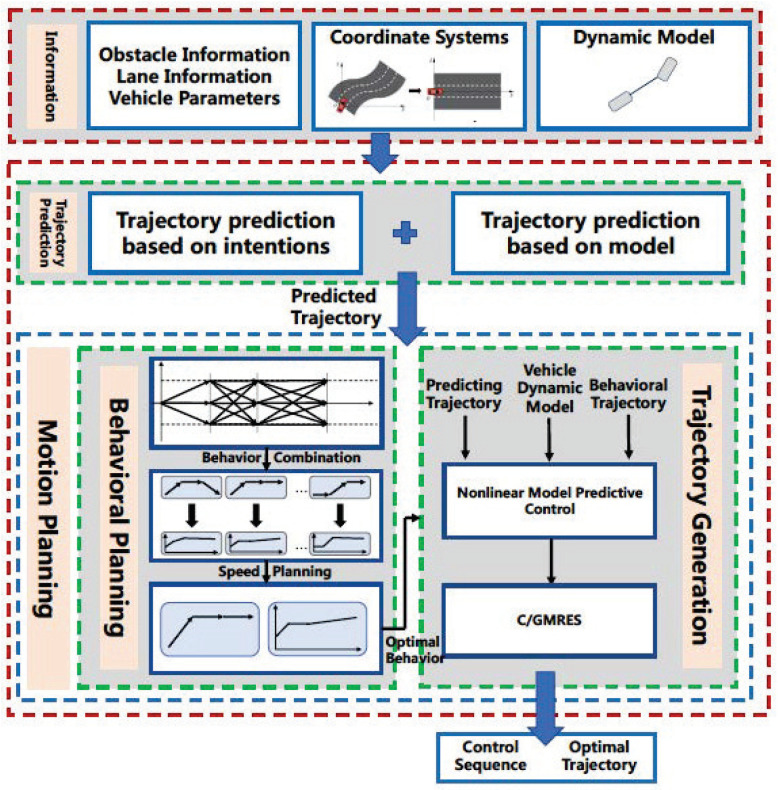
Diagram of the proposed motion planning frame.

As shown in Figure 1, this paper proposes a computational efficient motion planning method for autonomous vehicles, which can behave reasonably and adaptively in dynamic and complex driving scenarios. After predicting the trajectory of environment vehicles, the behavior planning and trajectory generation will be done sequentially. The following improvements simplify the problem and decrease calculation to realize computational efficiency. In the behavior planning module, the road is divided into several segments with road points and different behaviors are represented with different connections between road points in each segment. Firstly, rather than only set road points in the center of the lane, the road points are also distributed on the lane line, which enables the behavior planner to generate more complex behaviors. Meanwhile, in predictive control, short-term behavior is much more complex and also should be paid more attention to. Besides, the prediction is not accurate while lengthening the predictive horizon. Thus, rather than equally dividing the road, unequal segments that the distance between these road segments is gradually increasing can further decrease calculation and raise reasonability. Secondly, based on the analysis of different traffic participants, the motion of static environment vehicles is much more simple, and can directly exclude corresponding behaviors. Therefore, static environment vehicles are used to narrow the feasible region of the solution and further decrease online calculation. Thirdly, variational longitudinal velocity and dynamic vehicle models are considered to raise the reasonability of trajectory generation. It can also speed up the trajectory following process with the resulting acceleration and steering wheel angle. We use the C/GMRES algorithm to realize on-line calculations in NMPC. The main contributions are summarized as follows

- The complex and reasonable behavior of the host vehicle is efficiently realized by connecting different points located on unequally divided road segments and lane centerlines.
- Trajectory prediction of surrounding vehicles is considered during trajectory planning. And the trajectory planning is based on both driver’s intention and the kinematics vehicle model, which can increase the accuracy and rationality.
- C/GMRES is used to realize online calculation and raise the reasonability of trajectory generation and trajectory following.

The remainder of this paper is organized as follows. In Section 2, the coordinate systems and the trajectory prediction are introduced. Section 3 introduces the behavioral planning module. In Section 4, the trajectory generation module is introduced. In Section 5, the simulation process is shown, and the results are given and analyzed in detail. The simulation results verify the effectiveness of the proposed method. Section 6 is the conclusion of this paper.

## 2. Coordinate Systems Conversion and Trajectory Prediction

In this section, first, the conversion between the Cartesian coordinate system and the Frenet coordinate system is introduced to simplify the planning process. Then, three ways of trajectory prediction are illustrated and compared.

### 2.1. Coordinate Systems Conversion

**Figure 2:**
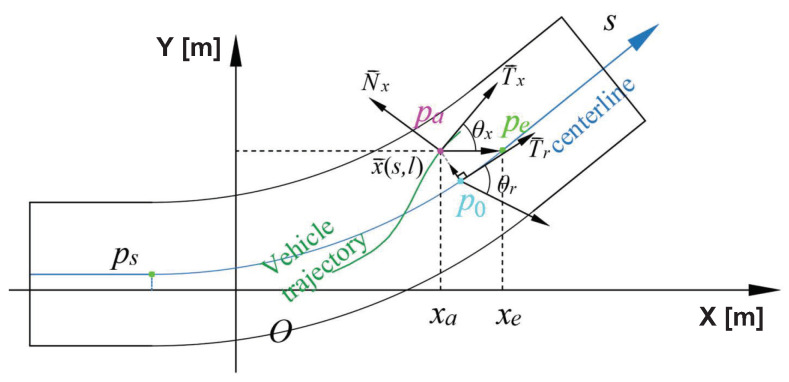
Diagram of coordinate systems conversion. $p_{s}$ is the starting point, $p_{e}$ is the ending point. $p_{a}$ is the point on the trajectory of the vehicle to be converted. $p_{0}$ is the nearest point that works as the reference point to convert $p_{a}$. ${\vec{T}}_{x}$ is the reference curve tangent vector in the Frenet coordinate system, and ${\vec{N}}_{x}$ is the normal vector in the Frenet coordinate system. $\vec{x}\left(s,l\right)$ is the Frenet coordinate of $\vec{x}$.

To describe the relation between two coordinates, as shown in Figure 2, the Cartesian coordinate of $\vec{x}$ is $\vec{x}\left(x,y\right)$ while the Frenet coordinate of $\vec{x}$ is $\vec{x}\left(s,l\right)$, where *l* is the distance from $\vec{x}$ to the reference point [24]. In coordinate systems conversion, the lane centerline is extracted with a third-degree polynomial equation as the reference curve of the Frenet coordinate system, e.g., $y=ax^{3}+bx^{2}+cx+d$. For the conversion from Frenet coordinate $C_{a}:\left(s_{a},l_{a}\right)$ to Cartesian coordinate $C_{a}:\left(x_{a},y_{a}\right)$, a nearest point $p_{0}$ works as the reference point to convert $p_{a}$, whose Frenet coordinate can be written as $C_{0}:\left(x_{0},ax_{0}^{3}+bx_{0}^{2}+cx_{0}+d\right)$. Since the arc length $s_{a}$ is already known, $x_{0}$ can be calculated by dividing the curve and sampling from the starting point $p_{s}$. The integral value of the sampling point $s_{0}^{\prime }$ can similarly be calculated. Compare $s_{0}^{\prime }$ with $s_{a}$ to determine whether the $x_{0}^{\prime }$ is the desired coordinate point $x_{0}$, and finally find the coordinates of $p_{0}$. And the Cartesian coordinate of $p_{a},C_{a}:\left(x_{a},y_{a}\right)$ can be calculated with

(1a) $$\begin{array}{cc} x_{a} & =x_{0}-l\cdot \sin\left(\arctan\left(k\right)\right)\;, \end{array}$$

(1b) $$\begin{array}{cc} y_{a} & =y_{0}-l\cdot \cos\left(\arctan\left(k\right)\right)\;. \end{array}$$

where $k=3ax_{0}^{2}+2bx_{0}+c$ is the curvature of the reference curve at point $p_{0}$.

For the conversion from Cartesian coordinate $C_{a}:\left(x_{a},y_{a}\right)$ to Frenet coordinate $C_{a}:\left(s_{a},l_{a}\right)$, the reference point $p_{0}$ is also needed. We use $D_{2}$ to represent the square of the distance from $p_{a}$ to the reference point $p_{0}$. The horizontal coordinate value of the reference point $x_{0}^{*}$ that minimizes $D_{2}$ satisfies $D_{2}^{\prime }\left(x_{0}^{*}\right)=0$, which can be solved by Newton’s method [25]. By calculating $D_{2}$ and $D_{2}^{\prime }$, the iteration formula can be expressed as

(2) $$\begin{array}{c} x_{0}^{*,m+1}=x_{0}^{*,m}-\frac{D_{2}^{\prime }\left(x_{0}^{*,m}\right)}{D_{2}^{\prime \prime }\left(x_{0}^{*,m}\right)},m=0,1,2,\ldots \;. \end{array}$$

The iteration stops while $|x_{0}^{*,m+1}-x_{0}^{*,m}|⩽\delta$ and $x_{0}^{*,m+1}$ is the target value. The Frenet coordinate $C_{a}:\left(s_{a},l_{a}\right)$ can be solved as

(3a) $$\begin{array}{cc} s_{a} & =\int_{x_{0}}^{x_{0}^{*,m+1}}\sqrt{1+{\left(3ax^{2}+2bx+c\right)}^{2}}dx\;, \end{array}$$

(3b) $$\begin{array}{cc} l_{a} & =D_{2}\left(x_{0}^{*,m+1}\right)=\sqrt{{\left(x_{a}-x_{0}^{*,m+1}\right)}^{2}+{\left(y_{a}-y_{0}^{*,m+1}\right)}^{2}}\;. \end{array}$$

### 2.2. Trajectory Prediction

The information about the surrounding vehicles and the trajectories of the surrounding vehicles in a period of time in the future is essential for motion planning. Such trajectory prediction can be done based on the driver’s intention, vehicle kinematics model, or both driver’s intention and vehicle kinematics model, which will be compared in this section.

#### 2.2.1. Trajectory Prediction Based on Driver’s Intention

We consider lane change and lane-keeping operations. For an operation intention, countless driving trajectories can be realized. Based on the driver of the vehicle, the actual driving trajectory may be very gentle or aggressive. In addition, the geometric environment of the road will also affect the actual trajectory. Therefore, the trajectory prediction based on the operation intention can generate a set of predicted trajectories based on the current state of the vehicle, the operation intention, and the road parameters, and then select the optimal one based on the information. Since the shape of the road has a great influence on the predicted trajectory, the predicted trajectory cluster is firstly generated in the Frenet coordinate system, then converted into the Cartesian coordinate system.

In Frenet coordinate system, s(t) and l(t) represent longitudinal distance and lateral distance, respectively. We use $F_{0}=\left(s_{0},{\dot{s}}_{0},{\ddot{s}}_{0},l_{0},{\dot{l}}_{0},{\ddot{l}}_{0}\right)$ and $F_{1}=\left(s_{1},{\dot{s}}_{1},{\ddot{s}}_{1},l_{1},{\dot{l}}_{1},{\ddot{l}}_{1}\right)$ to represent the initial state and the end state of the vehicle trajectory. To ensure the continuity of the trajectory and provide unique expressions for different trajectories, high-order polynomials are used for fitting to represent the trajectory of longitudinal s(t) and lateral distance l(t) over time *t*. For the initial state $F_{0}$, each state variable can be obtained by converting the current kinematic parameters in the Cartesian coordinate system, which can be expressed as

(4) $$\left\{\begin{array}{c} l_{0}=l_{0}^{*},\;{\dot{l}}_{0}=v_{0}\sin\left(\theta_{0}-\theta_{T_{0}}\right)\;,\\ {\ddot{l}}_{0}=\sqrt{\left(a_{0^{2}}+\gamma_{0}v_{0^{2}}\right)}\sin\left(\theta_{0}-\theta_{T_{0}}\right)\;,\\ s_{0}=0,\;{\dot{s}}_{0}=v_{0}\cos\left(\theta_{0}-\theta_{T_{0}}\right)\;,\\ {\ddot{s}}_{0}=\sqrt{\left(a_{0^{2}}+\gamma_{0}v_{0^{2}}\right)}\cos\left(\theta_{0}-\theta_{T_{0}}\right)\;, \end{array}\right.$$

where $l_{0}^{*}$ is the distance from the initial position to the centerline of the lane. $\theta_{T_{0}}$ is the orientation of the tangent vector ${\vec{T}}_{0}$. $\gamma_{0}v_{0}^{2}$ is the current value of the normal acceleration of the vehicle and γ is the curvature of the reference point.

We assume that the vehicle is on the center line of its target lane after finishing the intended operation and it remains the same longitudinal acceleration throughout the operation. Therefore, some of the operation termination state $F_{1}$ can be calculated as

(5) $$\left\{\begin{array}{c} l_{1}=l_{1}^{*},\;{\dot{l}}_{1}=0\;,\\ {\ddot{l}}_{1}=0,\;{\ddot{s}}_{1}=a_{0}\;. \end{array}\right.$$

$|l_{1}^{*}|=d$, where *d* is the width of the lane while the vehicle is changing the current lane and the sign is determined by the lane change direction. For lane keeping operation, $l_{1}^{*}=0$.

For a complete lane change operation, the time to complete the operation $t_{end}$ is about 6 s, and the length of $t_{end}$ can be adjusted according to the driver’s driving style. For lane-keeping operations, the time $t_{end}$ is significantly shorter. Use $t_{1}\in \left[0,t_{end}\right]$ to represent the end time of the operation, that is, take a fixed step size and sample start from 0 to $t_{end}$ with *K* steps. Since it is assumed that the longitudinal acceleration of the vehicle remains unchanged, the longitudinal speed at the termination state is ${\dot{s}}_{1}=v_{0}+a_{0}\cdot t_{1}$. For the lateral distance at the termination state, a fifth-degree polynomial fit for time *t* can be used, which is calculated as

(6) $$\begin{array}{c} l\left(t\right)=c_{5}t^{5}+c_{4}t^{4}+c_{3}t^{3}+c_{2}t^{2}+c_{1}t+c_{0}, \end{array}$$

where $c_{0},c_{2},\ldots ,c_{5}$ are the parameters of the curve. Then, $\dot{l}\left(t\right)$ and $\ddot{l}\left(t\right)$ can be calculated respectively. Therefore, the lateral state values at the initial state (t=0) and the terminal lateral state ($t=t_{1}$) can be written as

(7) $$\left\{\begin{array}{c} l_{0}=l\left(0\right)\;,{\dot{l}}_{0}=\dot{l}\left(0\right)\;,{\ddot{l}}_{0}=\ddot{l}\left(0\right)\;,\\ l_{1}=l\left(t_{1}\right)\;,{\dot{l}}_{1}=\dot{l}\left(t_{1}\right)\;,{\ddot{l}}_{1}=\ddot{l}\left(t_{1}\right)\;. \end{array}\right.$$

And the parameters $c_{i}$ can be obtained by solving the following matrix

(8) $$\begin{array}{cc} \left[\begin{array}{cccccc} 0 & 0 & 0 & 0 & 0 & 1\\ t_{1}^{5} & t_{1}^{4} & t_{1}^{3} & t_{1}^{2} & t_{1} & 1\\ 0 & 0 & 0 & 0 & 1 & 0\\ 5t_{1}^{4} & 4t_{1}^{3} & 3t_{1}^{2} & 2t_{1} & 1 & 0\\ 0 & 0 & 0 & 2 & 0 & 0\\ 20t_{1}^{3} & 12t_{1}^{2} & 6t_{1} & 2 & 0 & 0 \end{array}\right]\cdot \left[\begin{array}{c} c_{5}\\ c_{4}\\ c_{3}\\ c_{2}\\ c_{1}\\ c_{0} \end{array}\right] & =\left[\begin{array}{c} l_{0}\\ l_{1}\\ {\dot{l}}_{0}\\ {\dot{l}}_{1}\\ {\ddot{l}}_{0}\\ {\ddot{l}}_{1} \end{array}\right]\\ & =\left[\begin{array}{c} l_{0}^{*}\\ l_{1}\\ v_{0}\sin\left(\theta_{0}-\theta_{T_{0}}\right),\\ \sqrt{\left(a_{0^{2}}+\gamma_{0}v_{0^{2}}\right)}\sin\left(\theta_{0}-\theta_{T_{0}}\right),\\ 0\\ 0 \end{array}\right]\;. \end{array}$$

Since the longitudinal displacement changes according to $t_{1}$, a fourth-degree polynomial with respect to time *t* is used to fit the longitudinal kinematic, which can be expressed as

(9) $$\begin{array}{c} s\left(t\right)=c_{4}^{\prime }t^{4}+c_{3}^{\prime }t^{3}+c_{2}^{\prime }t^{2}+c_{1}^{\prime }t+c_{0}^{\prime }\;, \end{array}$$

where $c_{0}^{\prime },c_{2}^{\prime },\ldots ,c_{5}^{\prime }$ are the parameters. The initial states and the terminal states can be represented by replacing *t* in the above formula with 0 and $t_{1}$, respectively.

(10) $$\left\{\begin{array}{c} s_{0}=s\left(0\right)\;,{\dot{s}}_{0}=\dot{s}\left(0\right)\;,{\ddot{s}}_{0}=\ddot{s}\left(0\right)\;,\\ {\dot{s}}_{1}=\dot{s}\left(t_{1}\right)\;,{\ddot{s}}_{1}=\ddot{s}\left(t_{1}\right)\;. \end{array}\right.$$

Therefore, the parameters $c_{i}^{\prime }$ can be obtained by solving the following matrix

(11) $$\begin{array}{cc} \left[\begin{array}{ccccc} 0 & 0 & 0 & 0 & 1\\ 0 & 0 & 0 & 1 & 0\\ 4t_{1}^{3} & 3t_{1}^{2} & 2t_{1} & 1 & 0\\ 0 & 0 & 2 & 0 & 0\\ 12t_{1}^{2} & 6t_{1} & 2 & 0 & 0 \end{array}\right]\cdot \left[\begin{array}{c} c_{4}^{\prime }\\ c_{3}^{\prime }\\ c_{2}^{\prime }\\ c_{1}^{\prime }\\ c_{0}^{\prime } \end{array}\right] & =\left[\begin{array}{c} s_{0}\\ {\dot{s}}_{0}\\ {\dot{s}}_{1}\\ {\ddot{s}}_{0}\\ {\ddot{s}}_{1} \end{array}\right]\;,\\ & =\left[\begin{array}{c} 0\\ v_{0}\cos\left(\theta_{0}-\theta_{T_{0}}\right),\\ \sqrt{\left(a_{0^{2}}+\gamma_{0}v_{0^{2}}\right)}\cos\left(\theta_{0}-\theta_{T_{0}}\right),\\ v_{0}+a_{0}\cdot t_{1}\\ 0 \end{array}\right]\;. \end{array}$$

Different $t_{1}\in \left[0,t_{end}\right]$ corresponds to different fitting parameters *c* and different driving trajectories. When selecting the optimal trajectory, first convert the trajectory to the Cartesian coordinate system. The principles to be followed in the selection process include: $t_{1}$ is as short as possible, the driving process is comfortable, and the lateral displacement is reduced as much as possible during the lane change operation. Therefore, for the selection of the optimal predicted trajectory based on the driver’s intention, the maximum normal acceleration value in the driving trajectory and the time to complete the operation $t_{1}$ are mainly considered

(12) $$\begin{array}{c} C\left(traj_{i}\right)=w_{1}\max\left(\bar{a}\left(t\right)\right)+w_{2}t_{i}\;, \end{array}$$

where $traj_{i}$ represents the $i_{th}$ trajectory and $C(traj_{i})$ represents the cost of the $i_{th}$ trajectory. $\bar{a}\left(t\right)$ is the normal acceleration at time *t*. $t_{i}$ is the total time of the $i_{th}$ trajectory. $w_{1}$ and $w_{2}$ are two coefficients. The resulting trajectory with the smallest cost value is used as the optimal prediction trajectory based on the operation intention prediction

(13) $$\begin{array}{c} T_{\mathrm{man}}=\arg\;\min{\left(C\left(traj_{i}\right)\right)}_{i=1,\ldots ,K}\;. \end{array}$$

#### 2.2.2. Trajectory Prediction Based on Vehicle Kinematics Model

The trajectory predicted based on the driver’s intention is more accurate at a longer time horizon, but the accuracy is lower on a shorter time horizon. The trajectory predicted using the current kinematic parameters of the vehicle is more accurate in a shorter time. So, it is necessary to put both the intention and kinematics model into consideration. We assume that the vehicle acceleration and yaw rate remain unchanged. Therefore, in the Cartesian coordinate system, the vehicle speeds along the *x* and *y* axes at time *t* can be represented as

(14a) $$\begin{array}{cc} & v_{x}\left(t\right)=v\left(t\right)\cdot \cos\left(w_{0}t+\theta_{0}\right), \end{array}$$

(14b) $$\begin{array}{cc} & v_{y}\left(t\right)=v\left(t\right)\cdot \sin\left(w_{0}t+\theta_{0}\right), \end{array}$$

where the velocity at time *t* is $v\left(t\right)=a_{0}t+v_{0}$. The predicted trajectory based on the kinematic model can be obtained by integrating the vehicle velocities,

(15) $$traj_{mdl}:\left\{\begin{array}{c} x\left(t\right)=\frac{a_{0}}{w_{0}^{2}}\cos\left(\theta \left(t\right)\right)+\frac{v(t)}{w_{0}}\sin\left(\theta \left(t\right)\right)+c_{x}\;,\\ y\left(t\right)=\frac{a_{0}}{w_{0}^{2}}\sin\left(\theta \left(t\right)\right)-\frac{v(t)}{w_{0}}\cos\left(\theta \left(t\right)\right)+c_{y}\;, \end{array}\right.$$

where $c_{x}$ and $c_{y}$ are two parameters determined by the initial state and can be expressed as

(16a) $$\begin{array}{cc} & c_{x}=x_{0}-\frac{v_{0}}{w_{0}}\cos\left(\theta_{0}\right)-\frac{a_{0}}{w_{0}^{2}}\sin\left(\theta_{0}\right)\;, \end{array}$$

(16b) $$\begin{array}{cc} & c_{y}=y_{0}+\frac{v_{0}}{w_{0}}\sin\left(\theta_{0}\right)-\frac{a_{0}}{w_{0}^{2}}\cos\left(\theta_{0}\right)\;. \end{array}$$

In particular, when the initial yaw rate $w_{0}=0$, the predicted trajectory changes to

(17) $$traj_{mdl}:\left\{\begin{array}{c} x\left(t\right)=\left(\frac{1}{2}\cdot a_{0}\cdot t^{2}+v_{0}\right)\cos\left(\theta_{0}\right)+x_{0}\;,\\ y\left(t\right)=\left(\frac{1}{2}\cdot a_{0}\cdot t^{2}+v_{0}\right)\sin\left(\theta_{0}\right)+y_{0}\;. \end{array}\right.$$

#### 2.2.3. Trajectory Prediction Based on Both Driver’s Intention and Vehicle Kinematics Model

Since the predicted trajectory based on the kinematics model is more accurate only in a shorter prediction time, and the trajectory based on the driver’s intention has higher accuracy in a longer period of time, the predicted trajectory obtained by combining the two will be more accurate. Let the coefficient of the predicted trajectory based on the kinematics model be w(t), and the predicted trajectory model can be changed to

(18) $$\begin{array}{c} traj\left(t\right)=w\left(t\right)traj_{mdl}\left(t\right)+\left(1-w\left(t\right)\right)traj_{man}\left(t\right)\;, \end{array}$$

where w(t)∈[0,1] is a time-varying variable that is designed to predict trajectory with high accuracy. Here, w(t) is designed and tuned with multiple simulations as

(19) $$w\left(t\right)=\left\{\begin{array}{cc} \frac{2}{27}t^{3}-\frac{1}{3}t^{2}+1 & \mathrm{for}\;0\leq t<3\;,\\ 0 & \mathrm{for}\;t\geq 3\;. \end{array}\right.$$

**Figure 3:**
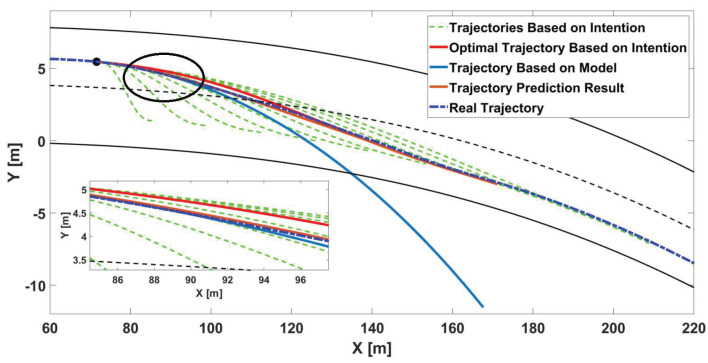
The results of trajectory prediction based on operation intention, vehicle kinematics model, and both operation intention and vehicle kinematics model.

Combined with the driver’s intention and the vehicle kinematics model, a more accurate predicted trajectory can be obtained. The results of trajectory prediction are shown in Figure 3. The trajectory that is predicted based on both operation intention and the vehicle kinematics model is more accurate than the other two methods.

## 3. Behavioral Planning

In the behavioral planning, by comprehensively considering the host vehicle information, road information, and surrounding environment information, an optimal behavioral trajectory is selected, which will be used for trajectory generation.

### 3.1. Generation of Candidate Paths

**Figure 4:**
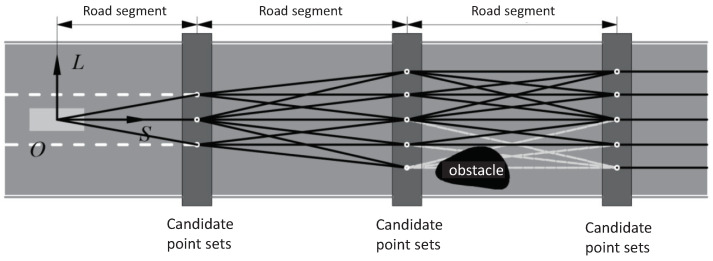
The diagram of Generation of Candidate Paths.

To reduce the size of the search space for trajectory planning and speed up the calculation, a series of candidate paths need to be generated first. The optimal path that does not collide is selected from the candidate paths. The candidate path is a candidate set that can represent the behavior that the vehicle can take in the planning process, and the road space of the entire prediction time is reasonably divided into multiple road segments. Comprehensively considering the prediction length and calculation time, the road space in the prediction time is divided into three road segments. The candidate points at the same *s* coordinates are called a layer of candidate point sets. The diagram of this division is shown in Figure 4. The road space occupied by each road segment can be represented as

(20) $$s_{i}=\left\{\begin{array}{cc} N_{i}\cdot \Delta s & \mathrm{for}\;v>v_{\min}\;,\\ N_{i}\cdot \Delta s_{min} & \mathrm{for}\;v\leq v_{\min}\;, \end{array}\right.$$

where Δs refers to the distance traveled by the vehicle in 1 s, $s_{i}$ refers to the length of the $i_{th}$ road space. To prevent the predicted length from being too short, a minimum interval $\Delta s_{min}$ needs to be set so that the vehicle can plan the trajectory even if the current vehicle speed is too slow. The value of $N_{i}$ is set based on the comprehensive consideration of the calculation time and the predicted length. The step length near the current position is short and the one farther away from the current position is large.

All candidate roads together constitute a candidate set of trajectories in the future. Each layer of candidate point sets contains the center point of both the current lane and the adjacent lane and the lane change point of the two lanes. By connecting the points in the candidate point set, a series of path candidate sets can be generated. To further narrow the search range, considering that the vehicle has a high risk of completing the lane change operation in a short period of time, the set of candidate points of the first layer contains only the road center point of the current lane and the lane change point with the adjacent lane.

The path candidate set is a connection of a series of points in the search space, but not every road can be driven in the path candidate set. There will inevitably be static and dynamic obstacles on the road. Regardless of the dynamic obstacles, the paths that pass through the static obstacles are firstly removed. When driving along these paths, the vehicle will inevitably collide with a static obstacle at any time. After removing, all paths in the remaining set of paths candidates will become candidate paths in the behavior decision layer. Speed planning is needed for these candidate paths to select the optimal one.

### 3.2. Speed Profile

**Figure 5:**
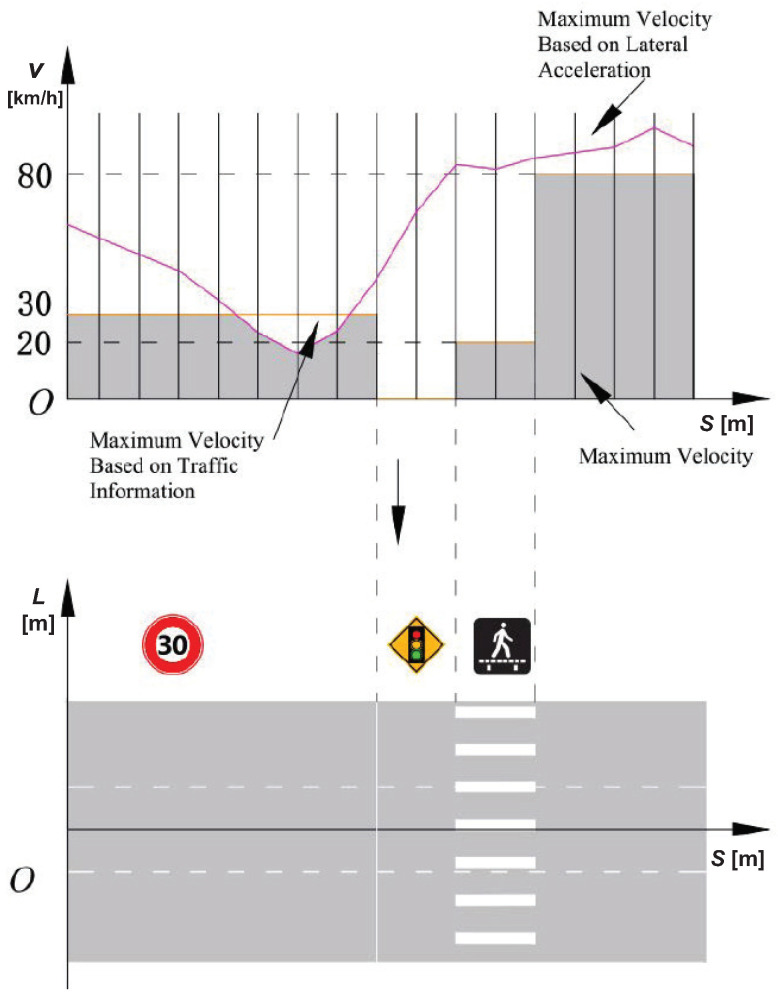
Maximum velocity considering traffic information and lateral acceleration.

The selection of vehicle speed needs comprehensive consideration of traffic and road information and restrictions, vehicle restrictions, dynamic obstacles, and other information. Traffic road information mainly includes traffic lights, traffic signs, stop lines, maximum and minimum speed limits, etc, which is simply shown in Figure 5. When selecting the optimal speed sequence, traffic road information must be extracted as the first constraint. Since the influence of road traffic information on vehicle speed mainly acts on the road driving direction, that is, the *S* direction, the limit curve based on the traffic road information on the vehicle speed can be represented in a *v*-*s* profile.

The maximum lateral acceleration of the vehicle while driving needs to consider the physical limitations of the vehicle and the impact on comfort. Based on the maximum lateral acceleration and the road curvature, the speed limit based on the lateral acceleration can be calculated as

(21) $$\begin{array}{c} v_{a_{y,max}}=\sqrt{\frac{a_{y,max}}{C_{Lane}}}\;. \end{array}$$

Since the road curvatures $C_{Lane}$ of adjacent lanes are the same, the limitation of the maximum lateral acceleration on the vehicle speed still only exists in the *S* direction. By comparing the value of the above two speeds, the limit curve of the vehicle speed in the *S* direction can be obtained as shown in Figure 5.

For dynamic obstacles, if time and space are considered at the same time, the problem of speed selection is a problem of optimal selection in *S*-*L*-*T* three-dimensional space, which is extremely high in calculation complexity. To reduce the dimension, each of the above candidate paths can be subjected to speed planning once. The coordinates *L* can be ignored to reduce the difficulty of calculation. An *S*-*T* profile is used to plan speed.

In an *S*-*T* profile, the *T* axis represents the time axis for predicting the future along the candidate road, and the *S* axis represents the space axis extending from the origin along the candidate road. We assume that the dynamic obstacles have constant acceleration within the prediction. The information on dynamic obstacles can be displayed in blue blocks and a safe distance is reserved too. The vehicle needs to travel in the space-time area corresponding to the blank grid. The origin of the *S*-*T* map is the current position of the host vehicle, and how to get to the target *S* position is the goal of speed planning.

Some restrictions and objective functions are necessary. First, the speed of the vehicle should be as fast as possible, which is calculated as

(22) $$\begin{array}{c} f_{v}=\left|v_{max}\left(s_{i}\right)-v_{i}\right|\;. \end{array}$$

In addition, large acceleration will reduce driving comfort, so acceleration needs to be limited as

(23) $$\begin{array}{c} f_{a}=\left|a_{i}\right|=\frac{|v_{i}-v_{i-1}|}{\Delta t}\;. \end{array}$$

Finally, it is necessary to avoid frequent acceleration and deceleration of the vehicle during driving, which is

(24) $$\begin{array}{c} f_{jerk}=\frac{|a_{i}-a_{i-1}|}{\Delta t}=\frac{|v_{i}-2v_{i-1}+v_{i-2}|}{\Delta t^{2}}\;. \end{array}$$

**Figure 6:**
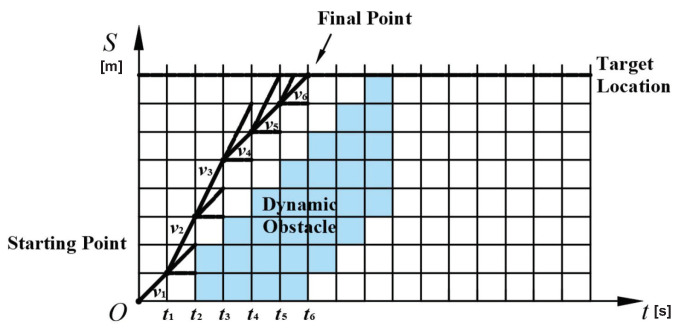
Speed profile.

In summary, the cost function is

(25) $$\begin{array}{c} f_{speed}=w_{v}f_{v}+w_{a}f_{a}+w_{j}f_{jerk}\;, \end{array}$$

where $w_{v}$, $w_{a}$ and $w_{j}$ are coefficients corresponding to $f_{v}$, $f_{a}$ and $f_{jerk}$. The optimal speed sequence for a certain behavior can be obtained through the cost function as shown in Figure 6.

### 3.3. Optimal Behavioral Trajectory Selection

Each candidate trajectory in the behavior planning candidate set represents a behavioral operation, and it is especially important to select the optimal one. It is necessary to consider efficiency, comfort, energy consumption, and other aspects.

First, the number of lane changes needs to be considered. $C_{LC}$ is a cost function factor affected by the number of lane changes. Since lane changing greatly increases energy consumption and greatly reduces comfort, it is necessary to avoid unnecessary and frequent lane changing operations. Thus,

(26) $$\begin{array}{c} C_{LC}=\sum_{k=1}^{N_{layer}}\left|Lane\left(n_{k}\right)-Lane\left(n_{k-1}\right)\right|\;, \end{array}$$

where $N_{layer}$ represents the layer number, $n_{k}$ represents the $k_{th}$ candidate point. $Lane(n_{\left(\cdot \right)})$ represents the lane at $n_{\left(\cdot \right)}$ candidate point.

In addition, since the *S* coordinate of the endpoint of each candidate route is the same, the shorter the travel time, the higher the efficiency. $C_{T}=T$ is only determined by the time. Frequent changes in behavior can reduce comfort and increase control difficulty. To reduce unnecessary changes in behavior planning, a consistency coefficient is introduced to indicate the difference between the candidate behaviors at the current time and the previously executed behavior. Thus, $C_{con}$ can be formulated as

(27) $$\begin{array}{c} C_{con}=\sum_{j=1}^{N_{b}-k}\left[{\left(S_{t}^{j}-S_{t-\Delta t}^{j+k}\right)}^{2}+{\left(L_{t}^{j}-L_{t-\Delta t}^{j+k}\right)}^{2}\right]\;. \end{array}$$

When generating the speed profile in the previous step, the candidate trajectory has been discretized. The step size after discretization is $\Delta t_{ST}$, and the total number of discrete points is $N_{b}=T/\Delta t_{ST}+1$. $\Delta t_{re}$ is the discrete step size of the model used for resampling performed. Then, $k=\Delta t_{re}/\Delta t_{ST}$. Therefore, $S_{t}^{j}$ and $L_{t}^{j}$ represent the coordinate values of the $j_{th}$ point on the candidate trajectory in Frenet coordinate system at time *t*. $S_{t-\Delta t}^{j+k}$ and $L_{t-\Delta t}^{j+k}$ represent the coordinate values of the $j_{th}$ point on the candidate trajectory in Frenet coordinate system at time *t*, which are the same corresponding point with $S_{t}^{j}$ and $L_{t}^{j}$. In this way, by constraining the difference between the same corresponding points in the two behavior planning paths taken at adjacent moments, the consistency and continuity of the behavior planning path can be constrained.

Last but not least, to make the vehicle location in the center of the road as much as possible while lane-keeping, $C_{boun}=\sum n_{boun}$ is used to represent the number of nodes where the vehicle is on the road boundary in the planned behavior trajectory, where $n_{boun}$ represents nodes where the vehicle is at the road boundary in the behavior trajectory.

In summary, a behavior path that is optimal in terms of efficiency, comfort, and energy consumption is selected according to the following cost function, and this behavior path is used as a reference for motion planning.

(28) $$\begin{array}{c} C\left(t\right)=w_{LC}C_{LC}\left(t\right)+w_{T}C_{T}\left(t\right)+w_{con}C_{con}\left(t\right)\;. \end{array}$$

### 3.4. Resampled Behavioral Trajectory

**Figure 7:**
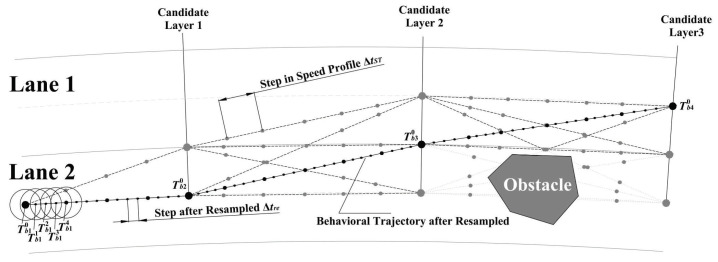
Resampled behavioral trajectory.

The step length of the behavior trajectory is longer. After selecting an optimal behavior trajectory, the trajectory needs to be converted from the Frenet coordinate system to the Cartesian coordinate system. The diagram of this process is shown in Figure 7. The step length of the candidate trajectory is $\Delta t_{ST}$, and the time sequence is $\Delta t_{ST}^{0}$, $\Delta t_{ST}^{1}$, …. The step size used in resampling is $\Delta t_{re}$, and the time sequence is $\Delta t_{re}^{0}$, $\Delta t_{re}^{1}$, …. Since $\Delta t_{ST}$ is larger than $\Delta t_{re}$, the optimal behavior trajectory needs to be interpolated to shorten the step size. In the process of interpolating, it is assumed that the vehicle speed remains unchanged within $\Delta t_{ST}$, and the number of $\Delta t_{re}$ contained in each $\Delta t_{ST}$ is $N=\Delta t_{ST}/\Delta t_{re}$. The time sequence of resampled trajectory can be written as $\Delta t_{ST}^{0}$, $\Delta t_{re}^{01}$, …, $\Delta t_{re}^{0(N-1)}$, $\Delta t_{ST}^{1}$, $\Delta t_{re}^{11}$, …, where $\Delta t_{ST}^{i}$ represents the time at the $i_{th}$ step, and $\Delta t_{re}^{ij}$ represents the $j_{th}$ resampled point in the $i_{th}$ step.

## 4. Trajectory Generation

With selected behavior from the behavioral planning module, nonlinear model predictive control is used in the trajectory generation module, which considers variational longitudinal velocity and dynamic vehicle model, and uses the C/GMRES algorithm to speed up the calculation process.

### 4.1. Vehicle Dynamic Model

To describe the vehicle dynamics characteristics more accurately, this paper uses a vehicle dynamics model. For the vehicle system, the coordinate system is the right-handed coordinate system, and the origin is the position of the center of mass of the vehicle. According to Newton’s second law, the dynamic characteristics can be represented as

(29a) $$\begin{array}{cc} 2(F_{yf}+F_{yr}) & =m\cdot a_{y}\;, \end{array}$$

(29b) $$\begin{array}{cc} \dot{w} & =2\frac{F_{yf}\cdot l_{f}-F_{yr}\cdot l_{r}}{I_{z}}\;, \end{array}$$

where *m* is the mass of the vehicle and $I_{z}$ is the inertia of the vehicle. $F_{yf}$ and $F_{yr}$ are the lateral forces of the front wheel and rear wheel, respectively. $a_{y}$ is the lateral acceleration. $l_{f}$ and $l_{r}$ are the lengths from the center of mass to the front axle and the rear axle, respectively. *w* is the yaw rate. β is used to represent the ratio of lateral speed to the longitudinal speed,

(30) $$\begin{array}{c} \beta =\frac{v_{y}}{v_{x}}\;. \end{array}$$

From the geometric relationship, the slip angles of the front and rear wheels can be represented as

(31a) $$\begin{array}{cc} \alpha_{f} & =\arctan\left(\frac{v_{y}+l_{f}w}{v_{x}}\right)\approx -\delta +\frac{wl_{f}}{v_{x}}+\beta \;, \end{array}$$

(31b) $$\begin{array}{cc} \alpha_{r} & =\arctan\left(\frac{v_{y}-l_{r}w}{v_{x}}\right)\approx \beta -\frac{wl_{r}}{v_{x}}+\beta \;. \end{array}$$

where δ is the steering angle. When the lip angle is small, the tire characteristics can be regarded as linear, which is calculated as

(32a) $$\begin{array}{cc} F_{yf} & =k_{f}\alpha_{yf}\;, \end{array}$$

(32b) $$\begin{array}{cc} F_{yr} & =k_{r}\alpha_{yr}\;. \end{array}$$

The derivative of longitudinal acceleration is the acceleration of the vehicle.

Using $X={\left[x,y,\phi ,v_{x},v_{y},w\right]}^{T}$ as the state vector of the vehicle dynamics system and $U={\left[a,\delta \right]}^{T}$ as the input vector of the vehicle dynamics system, the state equation of the vehicle dynamics system is

(33) $$\begin{array}{cc} \dot{X} & =f\left(X,U\right)=\left[\begin{array}{c} v_{x}\cos\varphi -v_{y}\sin\varphi \\ v_{x}\sin\varphi +v_{y}\cos\varphi \\ w\\ a\\ \frac{2k_{f}\left(v_{f}+wl_{f}-\delta v_{x}\right)+2k_{r}\left(v_{y}-wl_{r}\right)}{mv_{x}}-v_{x}w\\ \frac{2l_{r}k_{f}\left(v_{y}+wl_{f}-\delta v_{x}\right)-2l_{r}k_{r}\left(v_{y}-wl_{r}\right)}{I_{z}v_{x}} \end{array}\right]\;. \end{array}$$

Transform the state equation of the continuous form vehicle dynamics system into a discrete form

(34) $$\begin{array}{cc} & X_{k+1}=X_{k}+f\left(X_{k},U_{k}\right)\Delta t\;. \end{array}$$

### 4.2. Controller Design

To complete the task of trajectory planning, it is necessary to rationally design the objective functions and constraints of model predictive control. The input value of the vehicle system cannot exceed the limit of the physical structure, so the upper and lower limits of the input variable need to be limited as

(35a) $$\begin{array}{cc} & |a_{k}|\leq a_{max}\;, \end{array}$$

(35b) $$\begin{array}{cc} & |\delta_{k}|\leq \delta_{max}\;. \end{array}$$

To ensure that the planned trajectory does not collide with obstacles, it is necessary to maintain a certain safety distance between the trajectory at each moment and the obstacle at the corresponding moment. Since the longitudinal speed of the vehicle is often much higher than the lateral speed, a larger safe distance is needed in the longitudinal direction than in the lateral direction. The safety range around the host autonomous vehicle is designed as an ellipse, where the long axis of the ellipse is the longitudinal direction so that the safety constraint can be expressed as

(36) $$\begin{array}{c} {\left(\frac{x_{host}-x_{obs}}{r_{x}}\right)}^{2}+{\left(\frac{y_{host}-y_{obs}}{r_{y}}\right)}^{2}\geq 1\;, \end{array}$$

where $r_{x}$ and $r_{y}$ represent safety distance in the longitudinal direction and the lateral direction, respectively. The larger the safety distance is, the further the host vehicle acts.

To make the final trajectory planning result close to the behavioral planning path, the following objective function is needed

(37) $$\begin{array}{c} J_{coor}=w_{1}{\left(x_{k}-x_{ref,k}\right)}^{2}+w_{2}{\left(y_{k}-y_{ref,k}\right)}^{2}\;, \end{array}$$

where $x_{k}$ and $y_{k}$ are the coordinates of the trajectory at time-step *k*. $x_{ref,k}$ and $y_{ref,k}$ are the coordinates of the resampling behavioral planning path at time-step *k*.

As mentioned before, excessive acceleration and a small turning radius will greatly reduce driving comfort, and so it’s necessary to control their value of them.

(38) $$\begin{array}{c} J_{com}=w_{3}a_{k}^{2}+w_{4}\delta_{k}^{2}\;. \end{array}$$

A terminal constraint is added to ensure the final trajectory matches the planned behavioral path better.

(39) $$\begin{array}{c} J_{end}=\frac{1}{2}{\left(X_{N}-X_{ref,N}\right)}^{T}S_{f}\left(X_{N}-X_{ref,N}\right)\;. \end{array}$$

When there are two environment vehicles, the expression of the controller for motion planning is

(40a) $$\begin{array}{cc} \min_{\lambda_{M\times N}}J_{U_{i-N}} & =\frac{1}{2}{\left(X_{N}-X_{ref,N}\right)}^{T}S_{f}\left(X_{N}-X_{ref,N}\right)+\sum_{k=1}^{N}\left(w_{1}{\left(x_{k}-x_{ref,k}\right)}^{2}\right.\\ \; & \left.+w_{2}{\left(y_{k}-y_{ref,k}\right)}^{2}+w_{3}a_{k}^{2}+w_{4}\delta_{k}^{2}\right)\Delta t \end{array}$$

(40b) $$\begin{array}{cc} s.t. & {\dot{X}}_{k+1}=X_{k}+f\left(X_{k},U_{k}\right)\Delta t, \end{array}$$

(40c) $$\begin{array}{cc} & \left|a\right|\leq a_{max},\left|\delta \right|\leq \delta_{max}, \end{array}$$

(40d) $$\begin{array}{cc} & {\left(\frac{x_{k}-x_{obs,k}^{j}}{r_{x}}\right)}^{2}+{\left(\frac{y_{i}-y_{obs,k}^{j}}{r_{y}}\right)}^{2}\geq 1\;,j=1,2. \end{array}$$

Since the vehicle dynamics model used is a non-linear model, a suitable solving algorithm is necessary. In this paper, a Continuation/GMRES algorithm is used to solve the nonlinear model predictive control problem.

### 4.3. C-GMRES

In the nonlinear model predictive control problem, the small sampling period of mechanical systems will bring a great burden to the computing platform. Based on Generalized Minimum Residual Method (GMRES), Ohtsuka introduced the concept of the Continuation Generalized Minimum Residual Method (C/GMRES), which solves the linear equations involved in the differential equations at each sampling instant, thereby solving the control input sequence [26]. The detailed C/GMRES algorithm that is used in this paper is depicted in Algorithm 1.
**Algorithm 1** C/GMRES Algorithm*//Initialize t=0, l=0, initial state $x_{0}=x\left(0\right)$ and find $U_{0}$ analytically or numerically such that $||F(U_{0},x_{0},0)||\leq \delta$ for some positive δ, maximum iteration number $k_{max}$.*
1. For $t^{\prime }\in \left[t,t+\Delta t\right]$, compute the real control input by $u\left(t^{\prime }\right)=P_{0}\left(U_{l}\right)$.
2. At next sampling instant t+Δt, measure the state $x_{l+1}=x\left(t+\Delta t\right)$, set $\Delta x_{l}=x_{l+1}-x_{l}$.
3. $\dot{U_{l}}=FDGMRES\left(U_{l},x_{l},\Delta x_{l}/\Delta t,t,{\hat{\dot{U}}}_{l},h,k_{max}\right)$, where ${\hat{\dot{U}}}_{l}={\hat{\dot{U}}}_{l-1}$ with ${\hat{\dot{U}}}_{-1}=0$.
4. $U_{l+1}=U_{k}+{\hat{U}}_{k}+\Delta t$.
5.  Update t=t+Δt, k=k+1.


To solve the trajectory planning problem, first, the dummy inputs are used to convert inequality constraints into equality constraints.

(41a) $$\begin{array}{cc} & (\delta_{k}^{2}+u_{d1,k}^{2}-\delta_{max}^{2})/2=0\;, \end{array}$$

(41b) $$\begin{array}{cc} & (a_{k}^{2}+u_{d2,k}^{2}-a_{max}^{2})/2=0\;, \end{array}$$

(41c) $$\begin{array}{cc} & {\left(\frac{x_{k}-x_{obs,k}^{1}}{r_{x}}\right)}^{2}+{\left(\frac{y_{k}-y_{obs,k}^{1}}{r_{y}}\right)}^{2}-1-u_{d3,i}^{2}=0\;, \end{array}$$

(41d) $$\begin{array}{cc} & {\left(\frac{x_{k}-x_{obs,k}^{2}}{r_{x}}\right)}^{2}+{\left(\frac{y_{k}-y_{obs,k}^{2}}{r_{y}}\right)}^{2}-1-u_{d4,i}^{2}=0\;. \end{array}$$

Meanwhile, to prevent multiple solutions of dummy inputs, adding a small dummy penalty to the objective function

$$\begin{array}{cc} \min_{\lambda_{M\times N}}J_{U_{i-N}}= & \frac{1}{2}{\left(X_{N}-X_{ref,N}\right)}^{T}S_{f}\left(X_{N}-X_{ref,N}\right)+\sum_{k=1}^{N}\left(w_{1}{\left(x_{k}-x_{ref,k}\right)}^{2}\right.\\ & \;\left.+w_{2}{\left(y_{k}-y_{ref,k}\right)}^{2}+w_{3}a_{k}^{2}+w_{4}\delta_{k}^{2}-w_{U_{d}}U_{d,k}\right)\Delta t \end{array}$$

where $w_{U_{d}}$ is a small positive constant.

Then, define the Hamiltonian by

(42) $$\begin{array}{c} H\left(x,\lambda ,u,\mu ,p\right)=L\left(x,u,p\right)+\lambda^{T}f\left(x,u,p\right)+\mu^{T}C\left(x,u,p\right)\;, \end{array}$$

where $\lambda \in R^{n}$ represents costate and $\mu \in R^{m_{c}}$ represents language multiplier. $m_{c}$ represents the dimension of constraints.

For an optimal control ${\left\{u_{k}^{*}\right\}}_{k=0}^{N-1}$, it exists ${\left\{\lambda_{k}^{*}\right\}}_{k=0}^{N}$ and ${\left\{\mu_{k}^{*}\right\}}_{k=0}^{N-1}$, the following conditions should be satified

(43a) $$\begin{array}{cc} & x_{k+1}^{*}=x_{k}^{*}+f\left(x_{k}^{*},u_{k}^{*},p_{k}\right)\Delta t, \end{array}$$

(43b) $$\begin{array}{cc} & \lambda_{k}^{*}=\lambda_{k+1}^{*}+H_{x}^{T}\left(x_{k}^{*},\lambda_{k+1}^{*},u_{k}^{*},\mu_{k}^{*},p_{k}\right)\Delta t, \end{array}$$

(43c) $$\begin{array}{cc} & \lambda_{N}^{*}=\varphi_{x}^{T}\left(x_{N}^{*},p_{N}\right), \end{array}$$

(43d) $$\begin{array}{cc} & x_{k}^{*}=x\left(t_{0}\right), \end{array}$$

(43e) $$\begin{array}{cc} & H_{u}\left(x_{k}^{*},\lambda_{k+1}^{*},u_{k}^{*},\mu_{k}^{*},p_{k}\right)=0, \end{array}$$

(43f) $$\begin{array}{cc} & C(x_{k}^{*},u_{k}^{*},p_{k})=0. \end{array}$$

Here, Equaton (43) can be summarized as

(44) $$\begin{array}{cc} F(U(t),x(t),t) & =\left[\begin{array}{c} H_{u}^{T}\left(x_{0}^{*},\lambda_{1}^{*},u_{0}^{*},\mu_{0}^{*},p_{0}\right)\\ C(x_{0}^{*},u_{0}^{*},p_{0})\\ \ldots \\ H_{u}^{T}\left(x_{N-1}^{*},\lambda_{N}^{*},u_{N-1}^{*},\mu_{N-1}^{*},p_{N-1}\right)\\ C(x_{N-1}^{*},u_{N-1}^{*},p_{N-1}) \end{array}\right]\;,\\ & =0, \end{array}$$

Then, $\dot{F}\left(U,x,t\right)$ can be expressed as

(45) $$\begin{array}{c} \dot{F}\left(U,x,t\right)=A_{s}F\left(U,x,t\right)\;. \end{array}$$

Here, $A_{s}$ is an introduced stable matrix that stabilizes F(U,x,t) at the origin. Then, $\dot{U}$ can be computed with

(46) $$\begin{array}{c} \dot{U}=F_{U}^{-1}\left(A_{s}F-F_{x}\dot{x}-F_{t}\right)\;. \end{array}$$

The solution curve U(t) is approximated by forward difference if an initial solution U(0) satisfying F(U(0),x(0),0)=0 can be found. Here, generalized minimal residual (GMRES) method is applied to solve the linear equation $F_{U}\dot{U}=A_{s}F-F_{x}\dot{x}-F_{t}$. The combination of forward difference approximation and GMRES is called FDGMRES.

## 5. Simulation

In this section, the proposed computational efficient motion planning method is verified in three different environments. According to the information of the obstacle and the predicted trajectory, the behavior is selected, and the trajectory is optimized using NMPC, which is fast solved by the C/GMRES algorithm.

### 5.1. Obstacle Avoidance on Straight Lane

**Figure 8:**
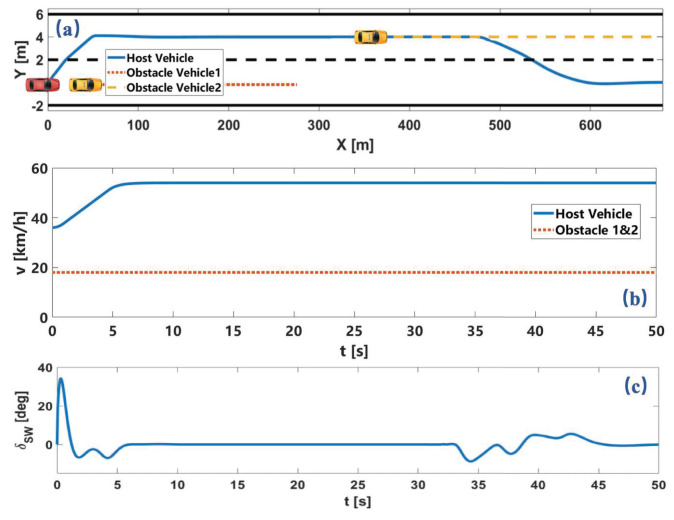
Simulation results of obstacle avoidance on a straight lane. Here, (**a**) is vehicle trajectory. (**b**) is vehicle velocity. (**c**) is steering wheel angle.

As shown in Figure 8, the host vehicle travels on a straight lane with an initial speed of 10 m/s. The maximum speed limit of the road is 15 m/s and each lane width is 4m. An obstacle vehicle is 30 m ahead of the host vehicle at speed of 5 m/s. The trajectory of the host vehicle and the obstacle vehicle is shown in Figure 8a. The speed profile of two vehicles and the steering wheel angle of the host vehicle is shown in Figure 8b,c. In this driving scenario, the host vehicle executes two consecutive lane-changing operations smoothly and quickly to avoid dynamic obstacles and keep the speed under the maximum speed.

**Figure 9:**
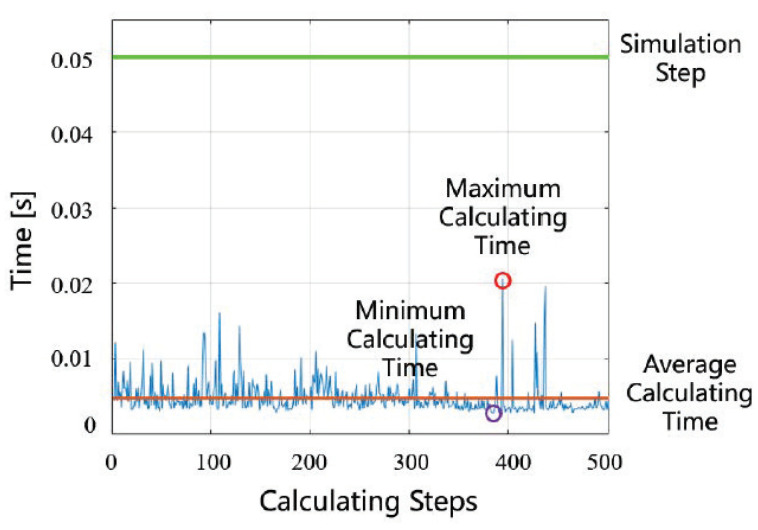
Calculating time.

The processor of the computer is Intel (R) Core (TM) i7-6700hq CPU@ 2.60GHZ. The time step size Δt for simulation is 0.05s and the nonlinear model predictive control problem using the C/GMRES algorithm, which is also compared with fmincon function in MATLAB. As shown in Figure 9, the calculating time of solving is much less than the time step size Δt, and also much less than the calculating time of the fmincon function in MATLAB which is more than 10 min. It shows that the local path planning module solved by C/GMRES can better meet the requirements of solving speed.

### 5.2. Obstacle Avoidance on Winding Lane

**Figure 10:**
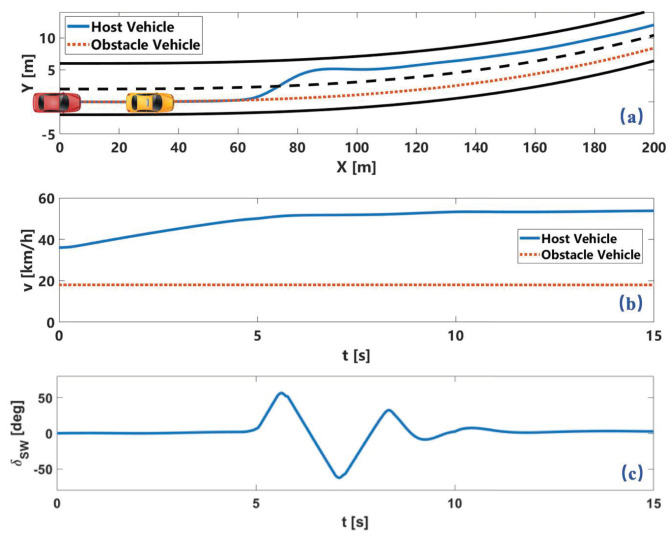
Simulation results of obstacle avoidance on winding lane. Here, (**a**) is vehicle trajectory. (**b**) is vehicle velocity. (**c**) is steering wheel angle.

In the scenario of a winding road, the center line of the initial lane is $y=10^{-6}x^{3}+10^{-5}x^{2}$ The obstacle vehicle travels at a speed of 5 m/s at 50 m in front of the host vehicle. The trajectory and speed profile of the two vehicles and the steering wheel angle of the host vehicle is shown in Figure 10. The host vehicle chooses to accelerate to overtake the preceding vehicle and avoid the obstacle.

### 5.3. Lane-Changing Obstacle Avoidance

**Figure 11:**
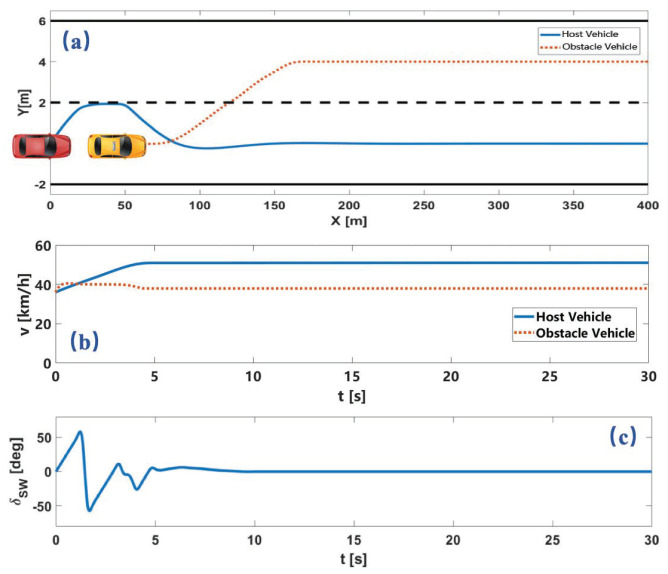
Simulation results of lane-changing obstacle avoidance. Here, (**a**) is vehicle trajectory. (**b**) is vehicle velocity. (**c**) is steering wheel angle.

A much more complex scenario is verified in the third simulation, in which the intention of the obstacle changed. The trajectory of the obstacle vehicle is selected from the open data set, which executes a lane change to the left lane. The trajectory and speed profile of the two vehicles and the steering wheel angle of the host vehicle is shown in Figure 11.

As shown in Figure 11a, at first, the obstacle vehicle chooses lane-keeping before changing lanes to the left lane. In this process, the host vehicle tries to change lanes. After the obstacle vehicle decides to change to the target lane of the host vehicle, the host vehicle decides to change back to its original lane. From the above simulation results, the proposed motion planning method considers safety, computational efficiency, and comfort simultaneously and obtains good system performance.

## 6. Conclusions

This paper proposes a computationally efficient motion planning method for autonomous vehicles, which considers dynamic obstacle avoidance and traffic interaction. The decision process for complex behavior is reasonably simplified in both time and space span. Different points located on unequally divided road segments and lane centerlines are connected to represent behavior. And C/GMRES algorithm is used to accelerate the calculation of the NMPC problem in the trajectory generation module. The trajectories of other traffic participants are more accurately predicted with known intention and vehicle models, which enables the movement to be more reasonably planned. Finally, three groups of simulation experiments are carried out to verify the rationality and superiority of the algorithm.

In future works, the interactive intention prediction will be considered in the intention predictive layer, which can extend the motion planning method from reacting adaptively to predicting adaptively. By considering the interactions between the ego vehicle and surrounding drivers socially via implicit and/or explicit communications, the behavior of the autonomous vehicle can be more human-like and facilitate safety performance under complex and dynamic environments [27]. 

## Data Availability

The data presented in this study are available on request from the corresponding author. The data are not publicly available due to privacy reason.
